# Demethylating agents in combination with CD7-targeted CAR-T for the successful treatment of a case with mixed-phenotype acute leukemia relapsed after allogeneic hematopoietic stem cell transplantation: A Case Report

**DOI:** 10.3389/fimmu.2023.1254010

**Published:** 2023-09-28

**Authors:** Ying Han, Hao Yao, Guang-cui He, Si-han Lai, Yan Deng, Shan Zhang, Ying He, Yi-song Xiong, Alex H. Chang, Yi Su, Hai Yi

**Affiliations:** ^1^ Department of Hematology, The General Hospital of Western Theater Command, PLA, Chengdu, China; ^2^ Department of Laboratory Medicine, The General Hospital of Western Theater Command, PLA, Chengdu, China; ^3^ Engineering Research Center of Gene Technology, Ministry of Education, Institute of Genetics, School of Life Sciences, Fudan University, Shanghai, China; ^4^ Shanghai YaKe Biotechnology Ltd., Shanghai, China

**Keywords:** mixed-phenotype acute leukemia, demethylating agent, CD7-targeted, chimeric antigen receptor-t cell therapy, allogeneic hematopoietic stem cell transplantation, demethylate

## Abstract

**Background:**

Allogeneic hematopoietic stem cell transplantation (allo-HSCT) has cured many patients with malignant hematologic diseases such as mixed phenotype acute leukemia (MPAL), while those relapsing after allo-HSCT still exhibit high mortality, poor prognosis, and no standard treatment modalities. It is necessary to explore more therapeutic modalities for patients with post-transplant relapse to obtain a better prognosis.

**Case presentation:**

In this case report, a young male with MPAL received allo-HSCT after reaching complete remission (CR) by induction chemotherapy. Unfortunately, relapse of both myeloid and T lineages occurred nine months later. After receiving demethylating chemotherapy, myeloid lineage measurable residual disease (MRD) turned negative. T-lineage MRD turned negative after CD7-targeted chimeric antigen receptor (CAR)-T cell therapy. The bone marrow remained MRD-negative for 4 months. This case preliminarily demonstrated a long-lasting CR with CD7-targeted CAR-T cell therapy, allowing a better prognosis.

**Conclusion:**

Demethylating drugs combined with CD7-targeted CAR-T cell therapy is feasible in treating MPAL patients with relapse after transplantation, with good efficacy and safety, which will be a promising treatment option for MPAL.

## Introduction

Allogeneic hematopoietic stem cell transplantation (allo-HSCT) is the preferred treatment method for mediate and high-risk acute leukemia. Post-transplantation relapse is one of the most important risk factors hindering the survival of patients after allo-HSCT. With recent medical developments, although the transplantation-related mortality rate has decreased, the risk of relapse remained the same. A study demonstrated that relapse-related mortality contributed 30% to 40% of all deaths post- allo-HSCT (1). Furthermore, patients who relapse after transplantation have a poor prognosis. Less than 20% of acute myeloid leukemia (AML) patients who relapse after allo-HSCT could achieve an overall survival (OS) of 2 years (2). There are various factors that cause relapse after transplantation, including disease biological features, risk stratification, pre-transplant disease status, donor type, and transplant complications (3). There is currently no standard treatment available for recurrence after transplantation. Common strategies consist of salvage chemotherapy, chimeric antigen receptor (CAR)-T cell therapy, donor lymphocyte infusion (DLI), and secondary HSCT (4). Therefore, it is necessary to explore more treatment methods to improve the prognosis of patients with post-transplant relapse.

CAR-T cell therapy is an effective immunotherapy developed and applied in B-cell tumors in recent years. CD19-targeted and CD22-targeted CAR-T cell therapies have shown high complete remission (CR) rates in relapsed/refractory (R/R) B-lymphocytic leukemia, diffuse large B-cell lymphoma and multiple myeloma. However, there is still a lack of approved CAR-T cell therapies for T-cell malignancies (5). CD7-CAR-T is an immunotherapy for T-lineage hematologic malignancies. As a transmembrane glycoprotein, CD7 is almost highly expressed in patients with both naive (T-acute lymphoblastic leukemia [T-ALL]/T-lymphoblastic leukemia/lymphoma [T-LBL]/natural killer [NK]/T-cell lymphoma) and mature (peripheral T-, NKT-, and mesenchymal large cell lymphoma) T-cell tumors, making CD7 an adequate target for cellular immunotherapy (6). Currently, there are several case reports and clinical trials of CD7-targeted CAR-T cell therapy for T-ALL, T-LBL and ETP-ALL/LBL (Early T-cell precursor acute lymphoblastic leukemia/lymphoma), showing encouraging good efficacy (7–9). However, further studies are needed for CD7-targeted CAR-T for T-ALL due to limited cases. We report a case of mixed phenotype acute leukemia (MPAL) recurrence after allo-HSCT receiving donor-derived CD7-targeted CAR-T cells combined with HMAs (demethylating agents) chemotherapy to assess the efficacy and safety of the approach. Our case report may provide a new therapeutic strategy among patients with post-transplant relapse of mixed phenotype acute leukemia (MPAL).

## Case presentation

A 32-year-old male patient was admitted to our hospital due to “intermittent lower abdominal pain for 20 days” on February 3rd, 2021. Based on result of cell blood count, blood smear, bone marrow smear, and flow cytometry (FCM) on bone marrow, he was considered MPAL. Later leukemia gene mutation tests confirmed *GATA3* 42%, *NOTCH1* 41%, *RUNX1* 39%, positive *JAK3* mutation. The leukemia fusion gene tested negative, chromosome: 46,XY, add(2)(p25) [19]/46,XY [1], clonal add(2p) was seen. The patient was then diagnosed to be MPAL. We started induction therapy on February 18th, 2021. DCAG (Dac+ACR+Ara-C+G-CSF) chemotherapy was given at first but not effective. Then we changed to the IA+L (IDA+Ara-C+PEG-ASP) regimen, and the patient received two sessions of chemotherapy. Measurable residual disease (MRD) and positron emission tomography/computed tomography (PET/CT) confirmed CR. The patient received haplo-identical transplantation from his daughter on June 16th, 2021. The preconditioning was as follows: The pretreatment regimen was total marrow and lymphoid irradiation (TML+I), TOMO (Tomo Therapy Hi Art) 200cGy twice a day from days -8 to -6, decitabine (DAC) 20 mg/m^2^ from days -5 to -3, cyclophosphamide (Cy) 1.8 g/m^2^ from days -5 to -4, cytarabine 2 g/m^2^ from days -5 to -4, as well as anti-thymocyte globulins (ATG) 7.5 mg/kg. After allo-HSCT, the patient’s bone marrow aspiration was monitored, no abnormalities were seen, and lumbar puncture and intrathecal injection were given regularly to prevent central system leukemia. As a follow-up maintenance regimen to prevent recurrence, venetoclax 100 mg daily (with oral itraconazole) and HDAC (histone deacetylase) inhibitor (chidamide) 5 mg 3 times/week were administered. The patient relapsed 9 months after transplantation on March 24th, 2022.

## Post-relapse treatment

The patient relapsed 9 months after transplant, and the bone marrow morphology showed 5% primitive cells. 6.52% of the cells in the FCM were malignant naïve T cells expressing CD7bri, CD99bri, CD5dim, CD34 + 1a; 2.07% of the cells were malignant naïve myeloid cells with T lineage expression, expressing CD117, cCD3, MPO, CD15, CD64, CD33, CD13. Meanwhile, PET/CT suggested that the new abnormal increase of fluorodeoxyglucose (FDG) metabolism in the mesenteric and retroperitoneum in the abdominopelvic cavity, posterior mediastinum parietal esophagus, multiple lymph nodes in the left clavicular region, spleen and prostate. The peripheral blood STR-T (daughter) is 98.91%. These indicated a relapse of leukemia. Donor-derived CD7-targeted CAR-T cell therapy was administered after getting consent from his family. Donor (his daughter) peripheral blood lymphocytes were collected and T cells were sorted. After chemotherapy and CD7-CAR-T treatment, the patient achieved remission. Specific disease progression and treatment are shown in **Table 1**.

**Table 1:** Disease progression and treatment of the case.

Time before and after CAR-T cell therapy/d	Date	Key events
-430	2021.2.8	Diagnosis of MPAL, T/myeloid (*GATA3*, *NOTCH1* , *RUNX1*, *JAK3* postive, chromosome 46,XY, add(2)(p25) [19]/46,XY)
-420	2021.2.18	1 cycle of DCAG chemotherapy (response evaluation: NR)
-395	2021.3.15	IA+L chemotherapy (response evaluation: CR)
-377	2021.4.2	IA+L chemotherapy (response evaluation: CR)
-311	2021.6.7	TMIL (2400cGy)+Dac+Ara-C+CY+ATG
-302	2021.6.16	allo-HSCT (6/10 HLA-matched related donor, daughter to father transplantation)
-246	2021.8.11	PTLD and accepted venetoclax+chidamide as maintenance therapy
-21	2022.3.24	6.25% of abnormal T cells , 2.07% of abnormal myloid cells (response evaluation: relapse), lymph node involvement
-14	2021.3.31	DAC+venetoclax+chidamide
-5	2022.4.9	Flu (30 mg/m^2^/day) and CTX (250 mg/m^2^/day) (response evaluation: CR)
-1	2022.4.13	1.82% of abnormal T cells, none of abnormal myloid cells
0	2022.4.14	CD7-CAR-T: 0.2 × 10^6^/kg
+21	2022.5.5	FCM: negative (response evaluation: CR)
+71	2022.6.24	FCM: negative (response evaluation: CR), none of swollen lymph nodes
+117	2022.8.9	FCM: negative (response evaluation: CR), none of swollen lymph nodes

**Figure 1:**
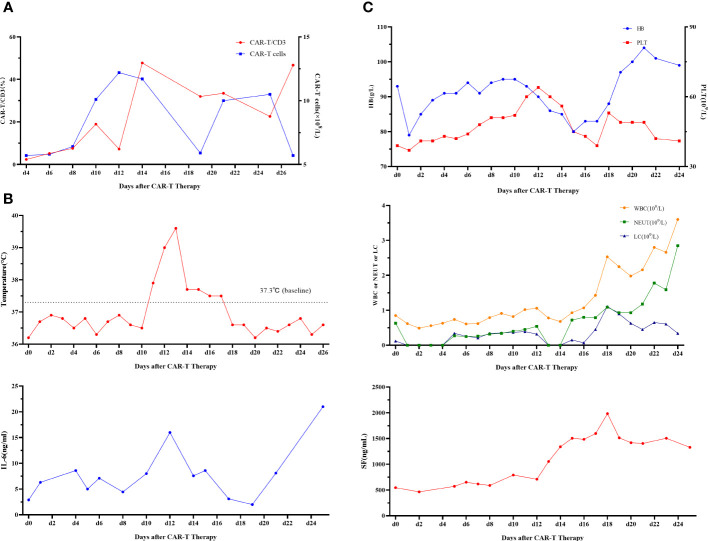
**(A)** CD7-CAR-T cells from peripheral blood. CAR, chimeric antigen receptor. **(B)** Change of each index. Changes in body temperature, WBC, neutrophil count, and lymphocyte count after CD7-CAR-T infusion. WBC, white blood cell; CAR, chimeric antigen receptor; IL, interleukin. **(C)** Changes in IL-6 and ferritin after CD7-CAR-T infusion.

**Figure 2:**
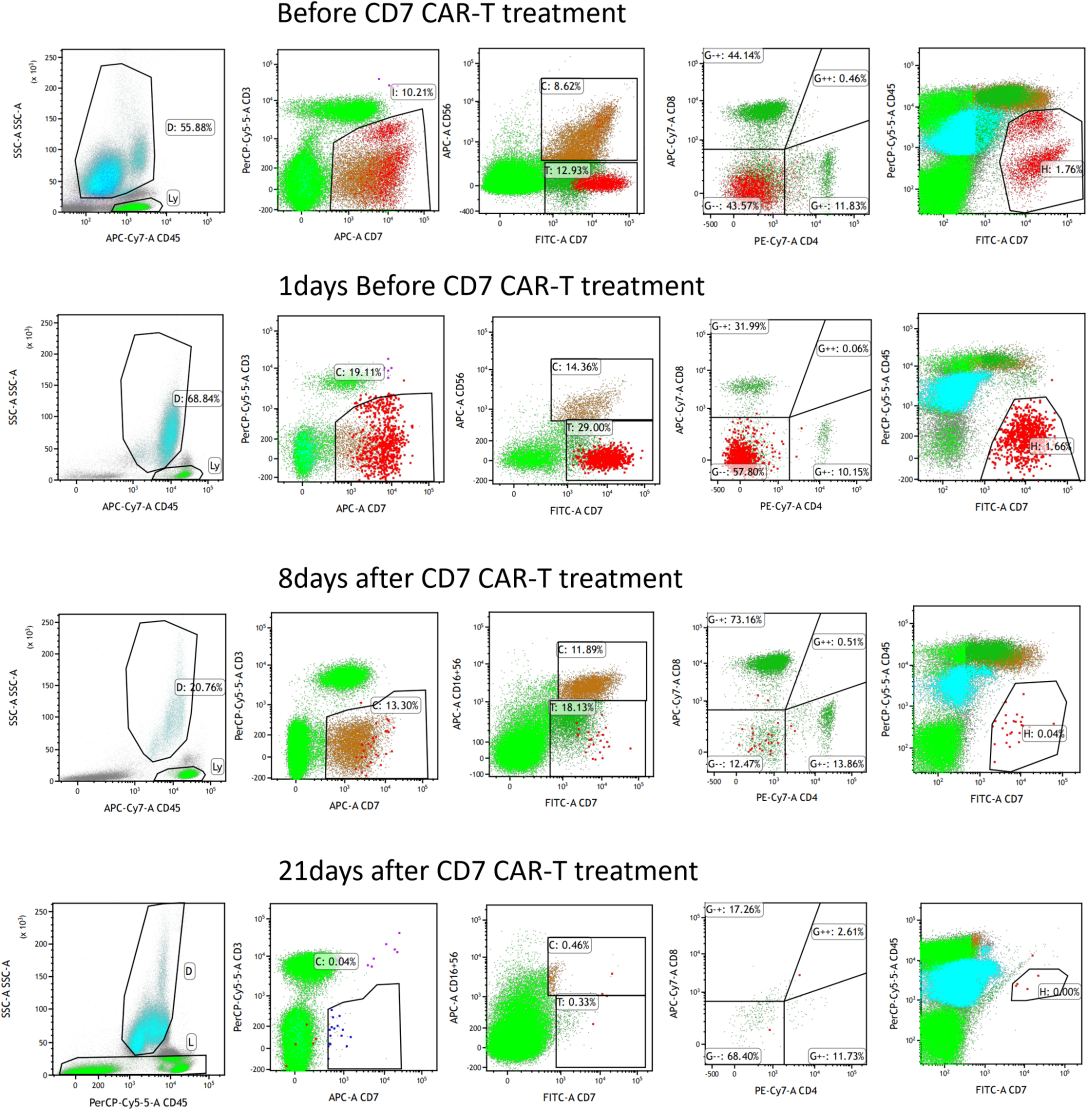
MRD was detected by flow cytometry after CD7 CAR-T therapy.

First, chemotherapy with decitabine 10mg on day -5 was administered to the patient on March 31, 2022, and both venetoclax and chidamide were discontinued upon completion of chemotherapy. CAR-T preconditioning with FC regimen (fludarabine 30 mg/m^2^ and Cy 30 mg/m^2^) from day 1 to day 3 was given on April 9. The bone marrow residual was examined again on April 13, 2022, showing 1.82% malignant naïve T cells and no naïve myeloid cells were detected. The patient’s myeloid MRD became negative following chemotherapy with decitabine, whereas T-series MRD remained positive. In order to turn T-lineage MRD negative, the patient received 0.2×10^6^/kg of CAR-T cells on April 14, 2022. On day +4 after CD7-CAR-T infusion, CAR-T cells started to amplify and reached a peak of CD7-CAR-T cells in peripheral blood on day +12, with the peripheral blood CAR-T/CD3 ratio of 12.2% and the absolute value of CAR-T cells of 7.24×10^8^/l (**Figure 1A**). From days +11 to +18, the patient experienced a grade I cytokine release storm (CRS), mainly manifested as fever with temperature fluctuations between 37.9°C and 39.6°C (**Figure 1B**). The patient did not have any other CRS reactions such as nausea, vomiting, weakness, headache, and muscle pain, so he was treated with dexamethasone(5mg×5d2.5mg×1d1mg×1d) immunosuppression and body temperature returned to normal. On day 14 after CD7-CAR-T cell infusion, the patient’s white blood cell (WBC), lymphocyte, and neutrophil counts dropped to a minimum. Then they gradually, reached their own peak on day 18 (2.53×10^9^g/l, 1.11×10^9^g/l, and 1.09×10^9^g/l, respectively) followed by a slight decrease and then continued to rise (**Figure 1B**). No significant changes were seen in hemoglobin (fluctuating from 80 to 100 g/l) and platelet count (fluctuating from 40×10^9^ to 60×10^9^g/l) throughout the course. In addition, interleukin (IL)-6 and ferritin were maintained at low levels throughout the infusion of CD7-CAR-T. Without the use of IL-6 antagonist (tocilizumab), a peak level of IL-6 of 21 ng/ml was observed on day 12, and ferritin peaked at 1984 ng/ml on day 18 after reinfusion (**Figures 1B, C**). A repeat bone marrow morphology on day +21 showed CR and negative MRD (**Figure 2**). Cerebrospinal fluid routine, biochemistry, and FCM did not show abnormalities. So far, four months following CAR-T treatment, the patient was in good condition, with a normal hemogram. As of August 9, 2022, (i.e., 117 days post-administration), 0.46% of CD7 CAR-T cells were still present in peripheral blood, as evidenced by the CAR-T/CD3 ratio. The recheck results of blood routine revealed a WBC count of 5.73×10^9^g/l, a lymphocyte count of 0.87×10^9^g/l, and a neutrophil count of 4.47×10^9^g/l. T-lymphocyte subsets revealed the levels of B lymphocytes (0.403×10^9^g/l), T4 lymphocytes (0.104×10^9^g/l), and T8 lymphocytes (0.37×10^9^g/l). On day +117, bone marrow morphology examination showed CR and negative MRD. The patient had no superficial lymph node enlargement, nor any plain or enhanced magnetic resonance imaging (MRI) abnormalities in the neck, shoulder, chest, pelvis, or abdomen. The patient has neither acute nor chronic graft-versus-host disease, nor any other fatal systemic adverse effects.

## Discussion

MPAL is a rare subtype of leukemia characterized by both lymphoid and myeloid immunophenotypic features, but the reason for the dual phenotype nature is unknown. It is unclear how this disease should be categorized, leading to uncertainty of MPAL treatment and poor prognosis (10). It was shown that adult MPAL patients who received HSCT after pretreatment of a myeloablative regimen by total body irradiation have a higher survival rate (11). In this case, we actively administered haploidentical transplantation in his first complete remission (CR1), and the patient still relapsed after surgery despite maintenance treatment with two targeted drugs, venetoclax and chidamide, which further confirmed the poor prognosis of MPAL. After treatment with decitabine, MRD was negative in his bone marrow, but still present in the T-lineage. Therefore, CD7-targeted CAR-T cell therapy was chosen. The patient experienced no severe CRS response during therapy and bone marrow MRD turned negative 21 days after infusion and sustained remission for 4 months with no extramedullary lymph node invasion. 117 days after infusion, the presence of CD7-targeted CAR-T cells was still detected in peripheral blood, indicating sustained CR and survival.

Several clinical trials and case reports have shown encouraging efficacy and manageable safety of CD7-targeted CAR-T in the treatment of R/R T-ALL/LBL, making CD7 an effective target for immunotherapy of T-cell malignancies (12). In a single-center phase I clinical trial featuring the Chinese population enrolled 20 patients with R/R T-ALL (CR rate, 90%), 10% of patients experienced grade 3-4 CRS and, the rest experienced grade 1-2 CRS (13). Another phase I clinical trial applying naturally selected CD7-targeted CAR (NS7CAR) products derived from patients or their transplant donors enrolled 20 patients with R/R T-ALL/T-LBL, demonstrating that NS7CAR-T cell therapy was a safe and highly effective treatment for T-ALL/LBL. These studies suggested that donor-derived CD7-targeted CAR-T cells could achieve high CR rates and manageable safety (14).

CD7-CAR-T infusion has the ability to effectively kill CD7+ T-ALL cell lines, which is an existing strategy for T-cell tumor-targeted therapy and worth further clinical investigation (15). However, the correlation between infusion of CD7-targeted CAR-T and possible subsequent secondary T-cell defects, as well as bridging grafts, urgently needs to be investigated. It has been suggested that CD7-targeted CAR-T infusion may deplete normal CD7-positive T cells and NK cells, leading to immunosuppression that would increase the risk of viral infection and latent virus reactivation (16). Hence, CD7-CAR-T cells can be the short-term treatment as a bridge to transplantation for patients with hematological diseases. Ke and Pan presented their latest study titled “Donor-derived CD7 CAR T Cells for patients with Relapse T-ALL after allo-HSCT: Phase I Trial” at the American Society of Clinical Oncology (ASCO) Annual Meeting, in which seven patients were enrolled with a 100% CR rate and an 85.7% 3-month CR rate, suggesting remarkable efficacy. However, 6/7 patients showed severe hematological toxicity after CD7-targeted CAR-T cell therapy and bone marrow hematopoietic recovery was slow, and secondary infections were common (17). Therefore, Ke et al. mentioned that recurrent T-ALL/LBL after HSCT requires secondary allo-HSCT as it will reduce the relapse rate and improve the prognosis. In this case, his hemogram started to recover (neutrophil count >0.5×10^9^/l) on day +18 after the infusion of CD7-CAR-T cells and the patient had no significant lung or viral infection during the 4-month follow-up period. We will continue to monitor his immune function status as the patient refused to undergo secondary HSCT.

In conclusion, CD7-targeted CAR-T cell therapy is a novel treatment option for T-ALL/LBL patients who relapsed after receiving HSCT, as well as an effective and safe treatment for relapsed patients bridging to secondary transplantation. For patients who are not eligible for secondary transplantation, whether CD7-targeted CAR-T cell therapy has long-term efficacy and whether it affects patients’ immune function will require long-term follow-up to assess its efficacy and safety. As a successful case with demethylating agents and CD7-targeted CAR-T cell therapy in one MPAL patient relapsed after transplantation, this case may serve as an important supplement for clinical application in China.

## Data availability statement

The original contributions presented in the study are included in the article/supplementary material. Further inquiries can be directed to the corresponding authors.

## Ethics statement

The studies involving humans were approved by The Ethics Committee of People’s Liberation Army The General Hospital of Western Theater Command. The studies were conducted in accordance with the local legislation and institutional requirements. The participants provided their written informed consent to participate in this study. Written informed consent was obtained from the individual(s) for the publication of any potentially identifiable images or data included in this article.

## Author contributions

YH: Data curation, Writing – original draft. HY: Funding acquisition, Writing – review & editing. GH: Project administration, Writing – review & editing. SL: Project administration, Writing – original draft. YD: Validation, Writing – original draft. SZ: Validation, Writing – original draft. YH: Data curation, Formal Analysis, Writing – original draft. YX: Conceptualization, Supervision, Writing – review & editing. YS: Writing – review & editing. HY: Conceptualization, Supervision, Writing – review & editing. AC: Conceptualization, Supervision, Resources, Writing – original draft.
